# Facilitators and barriers to volume management interventions in patients with heart failure and hyponatremia: a qualitative study

**DOI:** 10.1186/s12877-026-07080-y

**Published:** 2026-02-02

**Authors:** Hua Chen, Huiyan Chen, Yingxia Yao, Mengdie Liu, Xiaoyun Xiong

**Affiliations:** 1 The Second Affiliated Hospital of Nanchang University, No. 1 Minde/Mingde Road, Donghu District, Nanchang, 330006 China; 2 Nanchang University, Nanchang, China

**Keywords:** Heart failure, Hyponatremia, Volume management, Chronic disease, Qualitative research, Implementation science, Barriers and facilitators

## Abstract

**Background:**

Heart failure is highly prevalent among older adults, and volume management is central to both medical treatment and nursing care. Patients with concomitant hyponatremia often require tailored, hyponatremia-sensitive strategies; however, such personalized approaches are variably implemented in routine practice.

**Methods:**

Qualitative interviews guided by the Consolidated Framework for Implementation Research (CFIR 2.0) were conducted with 12 healthcare professionals, 10 patients with heart failure and hyponatremia, and 10 caregivers. Data collection continued until data saturation was reached, defined as no new themes emerging in three consecutive interviews. Data were analyzed using thematic analysis.

**Results:**

We identified three facilitator themes: (1) tailored targets and support that make volume management workable; (2) alignment of individualized targets with existing heart failure workflows; and (3) hyponatremia awareness and belief in the value of implementation. We also identified four barrier themes: (1) limited access to trusted, actionable evidence that undermines hyponatremia-sensitive volume management; (2) gaps in discharge preparation and care transitions that disrupt continuity across care settings; (3) awareness alone, without sufficient capability, that limits practical execution; and (4) geriatric constraints and limited support capacity that hinder sustained implementation.

**Conclusions:**

By synthesizing the identified facilitators and barriers, we found that volume management for patients with heart failure and hyponatremia represents a more individualized, dynamic, and precise approach than routine volume management. The barriers helped clarify current gaps in hyponatremia-sensitive volume management and informed multi-level strategies to address them. These findings provide a theoretical foundation for optimizing volume management in patients with heart failure complicated by hyponatremia in clinical practice.

**Trial registration:**

The trial was registered at the Chinese Clinical Trial Registry under the identifier ChiCTR2400089084 on September 2, 2024.

**Supplementary Information:**

The online version contains supplementary material available at 10.1186/s12877-026-07080-y.

## Introduction

Heart failure remains one of the major global health challenges in older adults (≥ 60 years) [1], affecting over 64 million individuals worldwide [2]. Volume management plays a pivotal role in heart failure management [2].Older adults with heart failure are particularly vulnerable to fluctuations in volume status due to age-related changes in renal function, hormonal regulation, making volume management even more critical in older adults [3].

Conventional low sodium and salt restriction advice is widely used to relieve congestion-related symptoms, yet hyponatremia (serum sodium < 135 mmol/L) occurs in a substantial proportion of patients in practice and is prognostically significant [4–6]. In patients with concomitant heart failure and hyponatremia, uniform sodium restriction may be inappropriate for some phenotypes and can conflict with concurrent fluid goals, underscoring the need for hyponatremia-sensitive, individualized sodium/fluid targets rather than blanket recommendations [7]. However, despite this clinical rationale, individualized hyponatremia-sensitive volume management remains suboptimal in real-world settings: practice is often anchored in generic low sodium messaging, non-standardized education, and inconsistent continuity across care settings, resulting in an implementation gap between recommended individualized management and routine delivery [8].

However, many patients with hyponatremia do not receive adequate individualized and precision volume-management interventions [9]. Fragmented transitions and poor documentation undermine continuity, and insufficient monitoring tools, patient or caregiver barriers, and resource pressures further reduce volume management [10]. Despite these clinical complexities, routine practice often continues to rely on standardized sodium-restriction protocols, with insufficient adaptation for hyponatremic subpopulations [11]. Moreover, volume management interventions are inconsistently delivered, hindered by gaps in clinical workflow, variable interdisciplinary coordination, and a lack of tailored patient education across settings [12]. Existing literature indicates that the implementation of volume management and hyponatremia-related interventions in heart failure is shaped by multilevel facilitators, including interdisciplinary collaboration, adaptable monitoring pathways, patient-engaged education, and persistent barriers, including limited operational guidance for hyponatremia phenotypes, fragmented workflows, insufficient caregiver support, yet concise synthesis of these determinants in real-world care remains limited [13].

Building on existing evidence on heart failure volume management [14–16], we conducted the Consolidated Framework for Implementation Research(CFIR 2.0) guided qualitative interviews to systematically identify multilevel determinants underlying the implementation gap in hyponatremia-sensitive volume management across care settings [17]. CFIR 2.0 is a determinant framework designed to identify multilevel facilitators and barriers that shape implementation across complex healthcare settings, making it well suited to our research question, which has been applied geriatrics, cardio-oncology service, and mental health [18–20].

Therefore, this study represents a formative, pre-implementation assessment intended to inform the subsequent design and adaptation of hyponatremia-sensitive volume-management strategies and implementation supports. Accordingly, this study aimed to explore the facilitators and barriers to implementing volume management interventions for patients with heart failure and hyponatremia across care settings, guided by CFIR 2.0 [17]. By integrating real-world perspectives from healthcare professionals and patients, this study seeks to inform the development of more context-specific, feasible, and patient-centered volume management strategies for patients with heart failure and hyponatremia.

## Methods

The datasets generated and analyzed during the current study not publicly but are available from the corresponding author upon reasonable request.

### Theoretical framework

CFIR 2.0 provides a comprehensive, multilevel lens to explore factors that influence intervention uptake, encompassing individual, organizational, and contextual domains [17]. Compared with its original version, CFIR 2.0 offers enhanced constructs such as patient needs and resources, health equity, adaptability, and team dynamics, which are highly relevant to complex care environments and chronic disease management [17].

Our study was guided by the updated CFIR 2.0 to systematically identify factors influencing the implementation of volume management interventions in patients with heart failure and hyponatremia [21]. CFIR2.0 is one of the most widely used implementation science frameworks, providing a structured approach to evaluate determinants of successful or failed intervention implementation across complex healthcare settings [22]. The framework comprises five major domains, including innovation, inner setting, outer setting, characteristics of Individuals, and process [23]. These domains are further organized into 48 constructs and 19 sub-constructs, allowing a nuanced and comprehensive analysis of the implementation environment [24]. The use of CFIR 2.0 in the pre-implementation phase is particularly valuable, as it enables researchers to anticipate modifiable barriers and identify local facilitators, which in turn inform necessary adaptations to the intervention or implementation strategies [25].

### Study design and setting

This qualitative study was conducted from May to July 2025 at the Second Affiliated Hospital of Nanchang University. The study aimed to explore perceived facilitators and barriers to volume-management interventions among patients with heart failure and hyponatremia, as well as the perspectives of healthcare professionals and family caregivers. We used semi-structured interviews to obtain in-depth, context-specific data from multiple stakeholders.

Participants included cardiologists, cardiovascular nurses, patients diagnosed with heart failure and hyponatremia, and their family caregivers. A combination of purposive sampling and maximum variation sampling was employed to ensure a diverse and information-rich participant pool. Physicians and nurses were selected based on variation in years of clinical experience, professional title, and administrative roles. For patients, sampling considered age, sex, years since heart failure diagnosis, and educational background. Interviews were audio-recorded, transcribed verbatim, and analyzed iteratively until data saturation was reached. Interviews were audio-recorded, transcribed verbatim, and analyzed iteratively until data saturation was reached, operationally defined as no emergence of novel themes after successive interviews [26]. Interviews and analysis were conducted iteratively and in parallel. Data collection continued until data saturation was reached. Saturation was assessed separately within each stakeholder group (healthcare professionals, patients, and family caregivers) and was operationally defined as no new codes and no substantive changes emerging in three consecutive interviews. To confirm stability, we conducted additional confirmatory interviews after the initial indication of saturation. Data saturation was achieved, ensuring finding enough barriers and facilitators faced by patients with heart failure and hyponatremia. The study outcomes were reported according to the consolidated criteria for the reporting of qualitative research(COREQ) [27].

### Inclusion and exclusion criteria

All patient participants were required to meet the following inclusion criteria: (1) were diagnosed with heart failure; (2) had at least one documented serum sodium level below 135 mmol/L; (3) older adults (≥ 60 years); (4) provided written informed consent. Exclusion criteria included: (1)communication disorders or inability to participate in the interview; (2) acute exacerbation; and (3)severe mental disorder and cognitive impairment. Eligible caregivers were those: (1) lived with the patient and were identified by the patient as the main caregiver; (2) had been actively involved in the patient’s care during the most recent month. All family caregivers also provided informed consent to participate in this study.

Healthcare professionals including doctors and nurses were contacted in advance to arrange interview times, and one-on-one interviews were conducted in a designated hospital meeting room. Doctors and nurses were eligible if they met the following inclusion criteria: (1) licensed healthcare professionals, cardiologists and nurses who have worked in the cardiology department; (2) had basic knowledge of volume management; (3) were available for at least a 30-minute interview; and (4)provided informed consent to participate in this study.

### Interview guide

A semi-structured interview guide was developed using the CFIR 2.0 to identify facilitators and barriers to implementing volume management interventions for patients with heart failure and hyponatremia. Following Damschroder’s recommendations, CFIR constructs were selected based on their relevance to the study context.

The interview guide was initially drafted based on a literature review and informed by the research team’s clinical experience and participants’ lived experience. During early interviews, we iteratively refined the guide using stakeholder input (clinicians, nurses, patients, and family caregivers) and team debriefings. Constructs that were repeatedly emphasized by stakeholders as most relevant to real-world implementation were retained and explored in greater depth in subsequent interviews, while less salient areas were de-emphasized.

The research team reviewed all CFIR 2.0 constructs and identified 11 constructs for patients and family caregivers and 18 constructs for doctors and nurses, covering the domains of innovation, inner setting, outer setting, characteristics of individuals, and process. These constructs informed the development of open-ended questions tailored to different stakeholder groups.

The interview guide(see the supplementary file) explored participants’ experiences, perceived challenges, and contextual factors related to volume management. All participants completed a brief demographic form, and patients provided additional information regarding heart failure history and self-management practices. Before interviews began, researchers explained the concept of volume management in heart failure with hyponatremia to ensure consistent understanding.

### Data collection and analysis

Interviews were conducted by three research team members (Chen Hua, Chen Huiyan, Yao Yingxia) based on an interview guide. The interview guide, coding manual, and coding methods were supervised and developed by experienced research team members (Xiong Xiaoyun, Chen Hua) in the field of implementation science and health services research. All interviews were face-to-face and conducted in Chinese. The duration of the interviews ranged from 15 to 30 min. The interview process was recorded, and audio files were automatically transcribed using iFlyTek software, followed by manual proofreading for verbatim transcription. After transcription and manual verification, all transcripts were de-identified by removing names and other potentially identifying information before being imported into NVivo for coding. NVivo software (QSR International, Melbourne, Australia) was used to manage and code qualitative data. Interviews were conducted until data saturation was reached, defined as the point at which no new themes emerged in three consecutive interviews. Saturation was monitored within each stakeholder group. The research team reviewed emerging codes after each interview and at regular intervals (every 2–3 interviews) through team debriefings, and recruitment continued 3 times for confirmatory interviews to verify stability before final stopping. Saturation was first reached after 9 interviews among healthcare professionals, 7 interviews among patients, and 6 interviews among family caregivers; the remaining interviews were conducted as confirmatory interviews.

We adopted a theory-driven thematic analysis following Braun and Clarke’s six-phase framework (2006) and applied a directed content analysis approach guided by CFIR 2.0 constructs. The transcribed text was analyzed through directed content analysis. A key theoretical framework, CFIR 2.0, was identified as the foundation, defining initial coding categories, which were validated, supplemented, and refined using textual data while identifying new categories not initially covered by the theory. An adjusted coding manual was constructed based on the interview guide, and the initial coding scheme draft was reviewed by the research team to reach consensus. All transcribed texts were coded by two members who regularly reviewed all codes to ensure consistency with the analysis framework. After the first round of coding, if necessary, the transcribed texts were re-read to confirm the coding. Subsequently, the team discussed the initial coding scheme, analyzed the interview quotes to propose optimization suggestions, making the coding more closely aligned with the data. Analysis results were submitted to the research team for review and further refinement to ensure compatibility with the data. Codes identified during the analysis process were organized into themes based on CFIR 2.0. For parts not represented in the data construction, we integrated or reorganized them to present the key elements of the data. This study adapt the Consolidated Criteria for Reporting Qualitative Research (COREQ).

To support figure-level synthesis and ensure theoretical alignment, we mapped each theme to CFIR 2.0 constructs and further categorized them a priori as “Facilitator,” “Barrier,” or “Mixed,” based on directional implications for implementation. Sensitivity checks were conducted to confirm coding consistency and directional patterns across coders without deviations. To enhance reflexivity, the research team documented analytic memos throughout the coding process and regularly discussed potential biases based on members’ disciplinary backgrounds.

### Minimize analytic bias and enhance rigor

To minimize analytic bias, transcripts were de-identified by removing names and other potentially identifying information prior to coding, and coding was conducted independently by two researchers and discrepancies were resolved through team discussion. Throughout analysis, we maintained an audit trail, including coding manual iterations, analytic memos, and consensus notes, and held regular reflexive meetings to examine how researchers’ clinical backgrounds and expectations might influence interpretation. Sensitivity checks were used to verify coding consistency and the stability of construct valence across coders.

### Research team and reflexivity

The research team consisted of qualified female nursing professionals, including three lead researchers and two trained assistants. The lead researchers were jointly responsible for study design, qualitative interviews, and data interpretation. All interviews were conducted collaboratively by two lead researchers—one serving as the primary interviewer, and the other providing support and managing recordings. Research assistants facilitated data organization and transcription. The third lead researcher reviewed analytic processes and adjudicated interpretive discrepancies to enhance analytic accuracy and reliability. All members received formal training in qualitative research methodology through the “Qualitative Research and Patient-Reported Health Outcomes Workshop” at Fudan University, China, and possessed substantial clinical nursing experience, ensuring methodological rigor and trustworthiness throughout the research process.

Interviews were conducted by three research team members (Chen Hua, Chen Huiyan, and Yao Yingxia) based on the interview guide. The interviewers were not involved in participants’ clinical care and had no prior relationship with participants.Their nursing backgrounds and clinical exposure to heart failure care supported effective communication with older patients and family caregivers, including using understandable language, pacing questions to reduce participant burden, and probing sensitively around hyponatremia- and volume-management experiences.The interview guide, coding manual, and coding methods were supervised and developed by experienced research team members (Xiong Xiaoyun, Chen Hua) in the field of implementation science and health services research, which supported methodological consistency during data collection and analysis. We also recognized that researchers’ clinical backgrounds and expectations could influence both data collection (e.g., emphasis during probing) and interpretation during analysis. To address this, the team held regular reflexive meetings and documented analytic memos to make assumptions explicit and examine potential bias during interpretation, alongside independent double-coding and consensus discussions.

### Ethical considerations

The study protocol was reviewed and approved by the Ethics Committee of the Second Affiliated Hospital of Nanchang University (Approval No. I-Medical Ethics Review [2024] No. (13)). This study was registered in the Chinese Clinical Trial Registry on September 2, 2024 (Registration No. ChiCTR2400089084). All procedures involving human participants were performed in accordance with the ethical standards of the institutional research committee and with the Declaration of Helsinki.

## Results

### Characteristics of participants

The study sample consisted of 32 individuals, including 12 healthcare professionals (3 cardiovascular experts, 3 doctors, and 6 nurses), 10 patients with heart failure and hyponatremia, and 10 family caregivers in Table 1. Specific characteristics of healthcare professionals and patients are presented in Tables 2 and 3, respectively.

**Table 1:** Interview participant characteristics

Participant	No.(%)
Healthcare professionals	12(37.50)
Patient	10(31.25)
Caregiver	10(31.25)

**Table 2:** Demographic characteristics of nurse participant and Doctor participant

Characteristic	Nurse Participant No.=6	Doctor Participant No.=6
Age, y, mean ± SD	30.2 ± 3.1	46.8 ± 14.8
Education, N(%)		
Secondary	0(0)	0(0)
Undergraduate	4(100)	1(16.67)
Postgraduate	0(0)	5(83.33)
Professional Title, N(%)		
Junior	3(50)	1(16.67)
Intermediate	3(50)	1(16.67)
Senior	0(0)	4(66.67)
Working Years, y,mean ± SD	7.2 ± 3.1	22.8 ± 16.4

**Table 3:** Demographic characteristics of patients participants

Characteristics	No.=10
Age, year, mean ± SD	77.7 ± 13.76
Sex, N(%)	
Male	7(70%)
Female	3(30%)
Education, N(%)	
Primary	1(10%)
Junior	1(10%)
Secondary	4(40%)
Associate degree	1(10%)
Undergraduate	3(30%)
Insurance Types, N(%)	
Resident medical insurance	4(40%)
Employee Medical Insurance	6(60%)
Number of hospitalizations in the past year, N(%)	
< 2	2(20%)
2–4	6(60%)
> 4	2(20%)
Caregiver Types, N(%)	
Children	4(40%)
Spouse	5(50%)
Professional Caregiver	1(10%)
Volume management education, N(%)	
Yes	4(40%)
No	6(60%)
Blood sodium level, N(%)	
< 125	2(20%)
125–130	4(40%)
< 135	4(40%)

### Facilitators and barriers

From both the perspective of healthcare workers, patients and family caregivers, we reported main facilitators and barriers related to implementing volume management interventions in patients with heart failure and hyponatremia and covered the domains of CFIR 2.0 (Tables 4 and 5).

**Table 4:** Description of subthemes by domain outlined in the updated consolidated framework for implementation Research (Patients and Caregivers)

Theme	Subtheme	Description	Example quotation
Innovation	Innovation source	The primary source of knowledge for patients and their caregivers comes from information provided by healthcare professionals. A small number of patients can obtain relevant information on volume management through mobile phones and other tools.(Facilitator)	C7: These are things that I only know because the doctor told me. P4:Mainly, when I was hospitalized before, the doctors and nurses at the hospital would tell me some things, and then I would also look up some information on public accounts and short videos myself.
	Complexity	Patients and their caregivers believe that the information provided within the hospital about capacity management is not detailed enough, and that online information is incomplete and lacks systematic organization.(Barrier)	C4: However, the discharge summary was too vague. It just said ‘low salt and low fat,’ but we don’t know how to eat that way. P6:I usually learn about health information through my phone, but I feel that the information is very scattered and there isn’t comprehensive guidance.
Individuals	Individual needs	Patients and caregivers have knowledge needs regarding capacity management.(Facilitator)	P5: I haven’t organized such activities that teach us how to measure our weight and exercise. I think it would be great if we did, as it would allow us to learn more and benefit our health. P7༚If possible, I would like doctors and nurses to call us more often to ask about our condition, and also organize some activities where we can learn practical knowledge to better take care of ourselves.
	Individual motivation	Patients and caregivers recognize the importance of capacity management and have a willingness to learn about related knowledge.(Facilitator)	C4: I think it’s quite important to do these things well, such as controlling water and salt intake, managing weight, taking medication on time, which can reduce the burden on his heart, lessen symptoms like chest tightness and dizziness, stabilize his condition, and prevent frequent hospitalizations. C6༚The doctor’s instructions must surely be for his own good, mainly to alleviate his condition. P2༚Although it conflicts with previous habits, for the sake of my own health, I can only gradually adapt. P8༚The community has never organized activities that teach us how to manage our daily lives. If such events were held, I might attend them.
Individuals	Individual ability	The patient’s capacity for self-management is inadequate, especially regarding medication usage and monitoring of their own symptoms. Caregivers focus on daily living care and have limited ability to assist patients with capacity management.(Barrier)	C5: At home, we mainly keep an eye on each other. Since I am also not in good health, I mainly remind him to pay attention to his diet, to eat less salt, less spicy food, and less oil, to control his water intake, and to monitor our weight changes together. When the time comes, I will also supervise him to have regular check-ups. P3༚I clearly know what medicines I need to take each day, but I don’t adjust the doses myself. I judge the effectiveness of the medication based on whether my body feels comfortable, though I can’t clearly describe the specific effects.
Inner setting	Networks and communications	Fragmented handoffs and inconsistent messages leave patients and caregivers unsure whom to contact, what to do, and when. Vague or conflicting discharge instructions, unanswered weekend calls, and unsynchronized EMR/WeChat threads undermine timely queries, threshold-based actions, and post-discharge continuity.(Barrier)	C2: There are no specialized tools at home for managing capacity, mainly relying on daily observation and care. C10༚There’s a blood pressure monitor to measure blood pressure, but my mom doesn’t know how to use it, so she always has to ask others for help. If possible, could the blood pressure monitor be more intelligent, making it easier for the elderly to operate? Or is there a set of equipment that can measure both blood pressure and weight at the same time, which would be more convenient?
	Compatibility	There is a lack of guidance on volume management for patients within the hospital and during follow-up after discharge. Additionally, there are issues with follow-ups not being timely.(Barrier)	P1: I was previously hospitalized at a county hospital. After being discharged, I did not receive any follow-up calls from the hospital nor did I have regular check-ups. This time, when I felt unwell, I came directly to a city hospital. P10༚I have received follow-up calls, which remind us to go for regular check-ups. They didn’t say anything else. C3: Previously, the hospital where we were admitted had us join a WeChat group, but when we had questions, no one responded to us. We still had to go to the hospital to get answers.
Outer setting	Local conditions	Inconvenient transportation can hinder patients’ timely access to medical care as well as their willingness to acquire knowledge about capacity management.(Barrier)	C3: Our home is in the county town, so every time we come here for medical treatment, it’s very troublesome. We have to borrow a car from relatives to get here, and the hospital in our county isn’t very good at treating this kind of illness. Plus, I feel more reassured about going to a big hospital. P1༚Although the county’s people’s hospital often has experts come down for free clinics, people find it troublesome and far away, so they don’t go out of their way to visit.
Outer setting	Local attitude	Patients and caregivers believe that primary care cannot provide sufficient assistance, and that primary hospitals do not adequately emphasize capacity management health education.(Barrier)	C5: Currently, the main channels for seeking help are hospitals. I feel that the community does not specifically provide assistance in this area, so when I have issues, I still rely on large hospitals. C7༚We won’t go to the community hospital because they don’t understand our situation. Since we have come here before, there must be records of us, and it will be more convenient for follow-up examinations. P3༚The community I live in has never organized such activities. I haven’t paid attention to events related to this either. Mainly, I just go to the hospital when there’s an issue. P9༚The community I live in has never organized such activities. I haven’t paid attention to events related to this either. Mainly, I just go to the hospital when there’s an issue.
Implementation process	Engaging	Proactive stakeholder engagement creates clear points of contact and rapid response channels, aligns roles for post-discharge follow-up, and sustains patient/caregiver participation in individualized, hyponatremia-aware volume management.(Barrier)	P4: The education was not hyponatremia-aware; I still did not understand how sodium monitoring should guide fluid restriction and diuretic use. C2: I was not formally engaged, yet I was expected to manage sodium-sensitive cooking and daily monitoring at home.
	Planning	Insufficient planning undermines individualized, hyponatremia-aware volume management: inconsistent needs assessment, absent thresholds and contingency steps, unscheduled 48–72 h follow-ups, unprepared devices/education, undefined cross-setting roles, missing checklists.(Barrier)	P2: Discharge planning lacked individualized sodium and fluid targets and clear hyponatremia-specific action steps. C3: The plan was not operationalizable for sodium restriction in mixed dishes, and practical estimation tools were missing.

**Table 5:** Description of subthemes by domain outlined in the updated consolidated framework for implementation research (Healthcare Professionals)

Domain	Subtheme	Description	Example quotation
Innovation	Evidence strength and quality	Clear, consistent, and practice-oriented evidence underpins a hyponatremia-aware, individualized volume-management strategy, increasing clinicians’ confidence, aligning teams on thresholds and workflows, and accelerating adoption at the bedside.(Facilitator)	D5: The latest heart-failure guidelines and multicenter trials give us strong justification to individualize sodium and fluid targets in hyponatremia—this evidence makes our decisions defensible and consistent. D2: Having protocol summaries that cite high-quality trials lets me titrate beyond generic “low-salt” rules and document why; it improves buy-in during rounds. N3: Our department pathway compiles the key evidence neatly, so I can translate it into concrete patient teaching and monitoring steps without hesitation.
	Innovation evidence base	Physicians use relevant guidelines and theories as the basis for volume management plans for heart failure with hyponatremia. The latest guideline updates provide direct evidence for comprehensive clinical assessment of patient conditions. But existing guideline evidence is insufficient in terms of specificity and detail and to be supplemented with clinical experience. (Mixed)	D2: All clinical measures are based on theory and guidelines. Guidelines provide inspiration for clinical work. We can learn from the latest guidelines and use them to adjust our clinical strategies and optimize the quality of our work. For example, the latest guidelines suggest that strict fluid restriction for heart failure patients is not advisable. Excessive fluid restriction may exacerbate hyponatremia, leading to symptoms such as fatigue, nausea, vomiting, or even confusion, and may worsen the condition and discomfort, affecting the patient’s quality of life. Therefore, in clinical practice, we should comprehensively assess the patient’s condition, and for patients with milder symptoms, we can appropriately relax the fluid restriction requirements. D4: Current guidelines lack specific details on fluid management for heart failure patients with hyponatremia, with no refined standards. Clinical practice relies more on individualized experience, such as the combined use of diuretics and sodium supplementation, or adjusting sodium restriction strategies based on the patient’s condition.
	Adaptability	Medical staff indicated that capacity management plans can be flexibly adjusted based on individual characteristics such as patients’ lifestyles, dietary habits, and economic status to enhance the feasibility of the plans. (Facilitator)	N4: Management measures primarily involve dietary guidance, tailored to the patient’s condition, to adjust sodium intake. control fluid intake, and for patients with heart failure stage IV, sodium intake should be kept below 2 g. However, when hyponatremia occurs, sodium intake should be appropriately increased. Additionally, the impact of diuretics on serum sodium levels should be assessed, and sodium-sparing diuretics should be selected. Once serum sodium levels return to the critical threshold, patients should be informed to resume sodium restriction. D4: Weight is a good indicator of the effectiveness of fluid management, is easy to implement, and has high patient acceptance. We can recommend that patients monitor their weight at home and adjust their diet based on weight changes. Emphasize home management, early identification of symptoms of disease progression (such as edema or dyspnea), and timely medical consultation.
	Compatibility	The volume management measures for patients with heart failure and hyponatremia are compatible with routine heart failure treatment and follow-up procedures, without disrupting existing medical models or increasing the burden on patients. (Facilitator)	N1: Upon admission, assess the patient’s cardiac function classification and exercise tolerance. Provide appropriate educational content based on the doctor’s treatment plan, including water and sodium restrictions, precautions for diuretic use, and the correct method for weighing. Record the patient’s weight and fluid intake/output daily during hospitalization. Prior to discharge, re-evaluate whether the patient’s water intake and sodium intake meet the required standards and assess the completion status. the patient’s subjective experience, and satisfaction levels. At discharge, provide health education, including monitoring and assessing edema, dynamic weight monitoring and warning signs, preparation of water and sodium restriction tools, and supervision and management of symptoms.
		The management plan for heart failure with hyponatremia is highly complex due to the nature of the disease, diverse patient needs, and home-based management. Challenges exist at the disease, intervention, and implementation levels, making clinical implementation challenging. (Barrier)	N5: I believe it is challenging to achieve a negative balance in fluid management to calculate the next day’s fluid intake and output plan, as it is difficult to determine specific, precise values. Patients have varying individual conditions, underlying diseases, and complex situations, resulting in significant individual differences, which require us to develop targeted fluid intake and output plans. D4: Individualized sodium management is challenging to achieve. Guidelines recommend a daily sodium intake of no more than 2 g, but this does not account for factors such as the patient’s cardiac function, use of diuretics, or whether they are in the decompensated phase, nor does it provide personalized, targeted sodium restriction plans or precise numerical values for sodium intake.
	Cost	Doctors say that the diuretic tolvaptan has little effect on sodium ions, but it is more expensive than other diuretics and is not used as a first-line treatment. (Barrier)	D2: Diuretics that have little effect on sodium ions, such as tolvaptan, are relatively expensive, placing a certain financial burden on patients. Therefore, they are not the first choice when selecting diuretics.
Individuals	Individual roles	Doctors and nurses clearly state their roles and responsibilities in capacity management. (Facilitator)	N1: I believe that nurses play the role of motivators, educators, and guides. First, they should train patients and their families in capacity management awareness and self-management skills, record daily weight measurements during hospitalization to monitor implementation effectiveness, and provide health education and remind patients to follow up after discharge. D6: Doctors are responsible for formulating treatment plans and evaluating and adjusting the effectiveness of subsequent plans.
	Individual cognition	Senior physicians possess professional knowledge regarding the hazards of diseases, management measures, guidelines, and individualized treatment, providing a scientific basis for the formulation of treatment plans. At the same time, there is a lack of awareness among young physicians regarding heart failure accompanied by hyponatremia. (Mixed)	D1: In clinical practice, I find that hypokalemia is more common than hyponatremia. I believe hypokalemia poses a greater risk, and in severe cases, it can be life-threatening. I pay less attention to hyponatremia in heart failure patients, and I have also observed fewer cases of heart failure accompanied by hyponatremia in my clinical work. This may be due to my limited clinical experience. D3: Heart failure may cause gastrointestinal congestion, leading to reduced intake and absorption, resulting in malnutrition; hyponatremia can cause fatigue in patients, affecting their compliance and adherence to heart failure rehabilitation.
	Individual ability	Doctors and nurses are highly skilled professionals, and their solid professional knowledge and clinical skills ensure the scientific validity of the program. (Facilitator)	N3: Patients with heart failure and hyponatremia have different sodium intake requirements than patients with ordinary heart failure. Therefore, when formulating measures, it is necessary to assess the patient’s cardiac function classification, the stage of heart failure, and whether the hyponatremia is caused by volume overload. Only after identifying the cause can the problem be solved at its source. D2: Develop personalized treatment plans based on the patient’s condition, including water and sodium restrictions, to ensure effective management and maximize relief of the patient’s discomfort from thirst, thereby improving patient compliance.
	Individual motivation	Medical staff improve patient outcomes, enhance clinical treatment standards, and improve patient experience through optimized treatment plans. This motivation stems from a sense of professional responsibility and professional pursuit. (Facilitator)	N4: Patients who use diuretics and restrict their water intake often experience significant thirst and typically choose to eat congee. However, congee is low in nutrients and can cause fluctuations in blood sugar levels. Therefore, we recommend eating steamed eggs or steamed fish to supplement protein and alleviate thirst. For patients with severe thirst, we also offer other relief methods, such as rinsing the mouth, sucking on mint, ice cubes, or dried plums.
	Knowledge and beliefs about the innovation	The complexity of the patient’s own disease, lifestyle habits, cognitive behavioral characteristics, and psychological factors directly or indirectly affect the understanding and adherence to the treatment plan, thereby increasing the difficulty of implementing the plan. (Barrier)	N4: Patients and their families have low levels of education and are unable to understand the importance of volume management; patients lack self-control, do not cooperate with medical staff, and their families are unable to correct their inappropriate behavior; for patients who have been hospitalized for an extended period, caregivers may experience caregiver fatigue and become desensitized to the patient’s non-cooperative behavior; regional differences and dietary habits may also contribute to poor patient compliance. D5: The challenge lies in post-discharge continuity of care, including how to control, monitor, and ensure the quality of the treatment plan at home. Reasons include entrenched daily habits that are difficult to correct; elderly patients living alone with poor self-management capabilities.
Inner setting	Acessibility of knowledge	Doctors and nurses can learn relevant knowledge through professional databases, academic journals, departmental study sessions, and other channels, with abundant opportunities and avenues for knowledge acquisition. (Facilitator)	N2: In terms of training, our department conducts regular professional development sessions, which include the latest guidelines and evidence-based summaries. These courses are typically taught by senior instructors to help us understand the latest trends. Additionally, new knowledge or common misconceptions are briefly discussed during morning handover meetings.
	Readiness for implementation	Incomplete organizational readiness limits consistent delivery of individualized, hyponatremia-aware volume management: protocols are not fully finalized or disseminated. (Barrier)	D3: We don’t have a finalized protocol with clear thresholds in the order set, so junior staff hesitate to adjust beyond generic low-salt advice. N4: Training was a one-off session; new nurses rotate in without standardized onboarding, and practice varies by shift.
	Patient needs and resources	When patients’ practical needs are explicitly addressed, such as clear low-literacy education, access to weighing scales/salt meters, easy transportation or tele-follow-up options, and family support, engagement and adherence to individualized volume-management increase. Matching resources to daily routines turns knowledge into action and sustains post-discharge self-management.(Facilitator)	N2: Our clinic’s WeChat group and short videos fit patients’ habits. When they understand what to do at home—how much to drink, when to call—we see better adherence. D4: With tele-follow-ups and weekend slots, patients from towns don’t delay visits. Meeting these access needs really improves continuity of volume management. D5: Providing a salt meter and a one-page pictorial guide helped families cook appropriately; caregivers now remind patients to log intake and we can titrate confidently.
Outer setting	External collaboration	Disconnect between hospitals and communities, insufficient support for capacity management from external collaborative relationships. (Barrier)	D3: Doctors have heavy clinical workloads, and follow-up tasks are time-consuming. The allocation of follow-up tasks is unreasonable and should be handled by community doctors or family doctors rather than specialists. A communication network should be established between hospitals, communities, and patients, incorporating general practitioners, community doctors, or primary care physicians to undertake follow-up and post-discharge patient management tasks, working collaboratively to jointly participate in patient capacity management.
Implementation process	Executing	Standardized, threshold-driven execution IO logging, auto-alerts for triggers, and rapid titration/slotting for follow-up—translates individualized, hyponatremia-aware plans into consistent day-to-day actions across ward, clinic, and home.(Facilitator)	D3: Once the weight crosses the trigger, we adjust diuretics and book a rapid review the same day; the protocol makes actions automatic. N4: Our EMR template mirrors the discharge sheet—patients log weight and intake/output in the app, and we get alerts to escalate or reassure. D2: Bedside teaching is copy-pasted into the home checklist, so patients and nurses follow the same steps—no gaps between ward and home.
	Reflecting and evaluating	Structured audit–feedback cycles convert data into rapid tweaks to thresholds, scripts, and EMR prompts. Sharing exemplars and fixing common pitfalls strengthens fidelity, shortens iteration cycles, and aligns teams on individualized, hyponatremia-aware practice.(Barrier)	D4: Our weekly dashboard flags missed sodium rechecks and late follow-ups; we adjust thresholds or scripts immediately and track impact the next week. N5: Audit emails with exemplar notes and common pitfalls improved documentation fidelity and helped us coach new staff more effectively.
	Reflection and evaluation	Medical staff pointed out that they would evaluate the effectiveness of the program through multidimensional indicators, and that the medical team would regularly conduct case discussions to analyze obstacles in the implementation of the program, summarize successful experiences, and optimize processes. (Facilitator)	N2: The quality control evaluation scale requires a relatively high level of knowledge from patients and their families. Relying solely on this evaluation scale may be too narrow and fail to adequately account for differences in the knowledge levels of patients and their families. It is advisable to incorporate images or other simple, easy-to-understand methods to help them comprehend and provide honest evaluations. Additionally, the frequency and duration of evaluations should be appropriately increased to enable dynamic management and assessment. N4: Doctors typically only record fluid output when prescribing orders, not fluid intake. Both fluid intake and output should be recorded to facilitate evaluation of the effectiveness of fluid management and the formulation of subsequent fluid management plans. D6: Doctors are responsible for developing treatment plans and evaluating and adjusting the effectiveness of subsequent implementation. I believe that clinicians have a good grasp of this knowledge. The key is to prioritize patient-centered care in daily work, pay attention to patients’ symptoms and feelings, and address their concerns. This aspect of work is crucial for the successful implementation of the entire treatment plan.

Patients and their family caregivers reported 3 constructs as the facilitators referring to innovation (innovation source), individuals (individual needs and individual motivation), and 8 constructs as barriers referring to innovation (complexity), individuals (individual ability), inner setting (networks and communications, compatibility), outer setting (local conditions, local attitude), and implementation process (engaging, planning).

Healthcare professionals reported 10 constructs as the facilitators referring to innovation(innovation source, adaptability, compatibility), individuals(individual roles, individual ability, individual motivation), inner setting (accessibility of knowledge, work foundation, available resources), and implementation process(adjust strategy, implementation and monitoring, reflection and evaluation), 6 constructs as barriers referring to innovation (complexity, cost), individuals (knowledge and beliefs about the innovation), inner setting(readiness for implementation), outer setting (external collaboration), and implementation process(reflecting and evaluating), and 2 constructs as the mixed factors including innovation (innovation evidence base) and individuals (individual cognition).

By further synthesizing these subthemes, we mainly identified the facilitators and barriers.

#### Facilitators

##### Tailored targets and support make volume management workable

Participants highlighted that volume management in patients with hyponatremia became more workable when targets and support were tailored and personalized to patients’ daily capacity, health literacy, and available family involvement. As shown in Tables 4 and 5, this facilitator mapped primarily to constructs related to self-management capacity, health literacy, and family involvement across stakeholder groups. Patients with heart failure described that rigid recommendations were difficult to follow when daily routines, comorbidities, or limited understanding were not adequately considered, especially combined with hyponatremia, which increased complexity and uncertainty to implement volume management. In contrast, guidance that was tailored to their daily context was perceived as more workable and easier to maintain.Family caregivers similarly emphasized the significance of suitable and appropriate support in sustaining volume management behaviors. These findings suggested that tailoring targets and support to patients’ everyday context may improve the feasibility and sustained engagement of volume management in real-world care. Notably, the perceived “fit” of tailoring differed by stakeholder role. Patients and caregivers emphasized day-to-day executability, such as whether targets match routines and available support, whereas clinicians focused more on whether personalization can be delivered efficiently within ward workflows.

##### Compatibility of individualized targets with existing heart failure workflows

Healthcare professionals emphasized that implementation was facilitated when hyponatremia-sensitive volume management could be embedded into routine heart failure care without disrupting existing clinical models or adding undue burden. As summarized in Tables 4 and 5, this facilitator was primarily reflected in the Compatibility construct, alongside related workflow-oriented elements. Clinicians described that integrating individualized targets for patients with hyponatremia into standard admission and inpatient workflows enabled more consistent delivery. Participants reported that integrating standardized documentation and patient-facing tools into routine discharge workflows strengthened continuity across settings. Specifically, discharge-aligned electronic medical record templates and app-based intake and output logging with alert functions, coupled with transferring bedside education into a home checklist, helped ensure consistent guidance and follow-up from hospital to home. Taken together, these findings suggested that integrating individualized, hyponatremia-sensitive targets into established heart failure workflows may support more consistent volume management across the inpatient-to-home transition.

##### Hyponatremia awareness and belief promote the implementation of volume management

Nearly all participants acknowledged the importance of receiving appropriate volume management interventions when hyponatremia is present. Patients expressed a strong belief that trusted volume management recommendations would increase their adherence, subsequently improving their overall health and reducing hospitalizations.

Cardiologists demonstrated great familiarity with recent volume management in patients with heart failure and hyponatremia and expressed confidence in its potential to reduce hospitalizations and improve heart failure symptoms.

#### Barriers

##### Limited access to trusted and actionable evidence undermines hyponatremia-sensitive volume management

Participants described that implementation was hindered when credible, operationalizable information on hyponatremia-sensitive volume management was not readily available, resulting in unreliable patient education and variable clinical decision-making. As shown in Tables 4 and 5, patients and caregivers reported that discharge education about volume management in patients with heart failure and hyponatremia was not detailed enough and that online information was scattered and unsystematic, which was far from being detailed and reliable during hospitalization. Clinicians reported that current guidance lacked refined, actionable standards for fluid management in heart failure with hyponatremia. Patients and caregivers noted that discharge instructions were often too vague to translate into daily practice, such as “low salt and low fat” without clear and specific steps, which contributed to uncertainty when managing sodium decisions at home. Clinicians similarly emphasized that while guidelines and trials provide direction, the insufficient specificity in existing evidence means practice frequently relies on individual experience. Importantly, what counts as “usable evidence” appeared to vary across groups: clinicians sought operational standards and decision triggers, while patients and caregivers needed concrete, comprehensible steps for meals and symptom responses. This discrepancy may explain why the literature often verifies consistency at the guideline level, yet real-world practice diverges.These findings suggested that gaps in trusted education materials and actionable evidence reduced confidence and consistency in implementing hyponatremia-sensitive volume management across care settings.

##### Gaps in discharge preparation and care transitions disrupt continuity across settings

Participants reported that implementation was hindered when discharge preparation and cross-setting transitions were insufficient, leading to fragmented support from hospital to home and limited linkage with community follow-up. This barrier was evident in patient reports of limited post-discharge contact, including the absence of follow-up calls and routine reviews, as well as follow-up interactions that were largely limited to reminders for check-ups without additional, actionable guidance. Caregivers likewise reported that available communication channels did not consistently provide timely support; for example, one caregiver noted that although they were invited to join a WeChat group, questions raised in the group often went unanswered. Clinicians reported that discharge materials often lacked actionable plans, and nurses noted that early follow-up was inconsistently routinized, depending on duty staff and leaving some patients unscheduled, although individualized volume management within the hospital was performed adequately. These findings suggested that gaps in discharge planning and care transitions can disrupt continuity and undermine sustained implementation of hyponatremia-sensitive volume management from hospital to home.

##### Awareness alone, coupled with insufficient capability limits practical volume management execution

Although awareness in volume management for patients with heart failure and hyponatremia was present, participants described gaps in practical skills and execution. Nearly all participants expressed awareness of the importance of volume management and a willingness to follow recommendations. However, as shown in Tables 4 and 5, this awareness that translated into actionable self-management capability was difficult. Patients and caregivers reported confusion and unease when dietary guidance shifted. Patients were initially instructed to follow a low-sodium diet but were sometimes later advised to increase salt intake, making inconsistent standards difficult to reconcile with established eating habits. Patients also described difficulty translating knowledge into clinical decision-making. Patients noted that although they knew which medications to take daily, they did not adjust doses independently and judged effectiveness primarily based on general comfort rather than specific symptom indicators. Clinicians similarly observed that low educational attainment, reduced comprehension, and self-regulation challenges constrained patients’ ability to implement recommendations consistently. These findings suggested that cognition and perceived importance were present, yet implementation remained limited by insufficient skills, and day-to-day execution capacity.

##### Geriatric constraints and limited support capacity hinder sustained hyponatremia-sensitive volume management

Participants described that volume management implementation was hindered by geriatric-specific limitations, including advanced age, reduced comprehension, functional constraints, and difficulty using monitoring tools. As shown in Table 3, the sample was predominantly older. Clinicians further noted that lower educational attainment and reduced understanding constrained older patients’ ability to grasp and consistently apply recommendations, and that challenges such as low self-control, entrenched habits, and living alone could weaken post-discharge adherence. Caregivers highlighted age-related barriers to home monitoring, reporting that although devices might be available, but the elderly can’t use it without others’ help. Caregiver participants also described functional and access constraints that disproportionately affect older adults, including transportation difficulties that limited follow-up and the ability to learn and reinforce volume-management skills after discharge, alongside insufficient community-based support for ongoing guidance. These findings suggested that age-related cognitive and functional limitations, compounded by restricted access to education and support, undermined sustained implementation of hyponatremia-sensitive volume management from hospital to home.

## Discussion

This qualitative study explored perceived facilitators and barriers to implementing and sustaining volume management for patients with heart failure and hyponatremia in a tertiary hospital in China. CFIR-based systematic coding identified that the influences on patients’ volume management could be categorized into multiple dimensions including innovation, outer setting, inner setting, individuals, and implementation process. These factors identified the complexity of volume management in patients with heart failure and hyponatremia. Key facilitators included tailored targets and support, workflow compatibility and embedding of individualized targets into routine heart failure care, and hyponatremia awareness and belief in the value of volume management. Barriers included limited access to trusted and actionable evidence, gaps in discharge preparation and care transitions, awareness without sufficient capability to execute volume management in practice, and geriatric constraints and limited support capacity.

### Facilitators: beyond perceived feasibility, also higher requirements

In this study, the identified facilitators suggested not only perceived feasibility but also higher requirements. Participants’ accounts tended to frame hyponatremia-sensitive volume management as a more personalized, refined, and dynamic extension of routine heart failure volume-management principles, described as requiring greater precision, ongoing monitoring, and clear communication, rather than as an approach that negates or stands in opposition to conventional practice. Similarly, this framing echoes diabetes care, where clinicians balance hyperglycaemia control with hypoglycaemia prevention [28]. Drawing on the facilitators identified (tailored targets and support, workflow compatibility, and hyponatremia awareness and belief), participants generally perceived that advancing “hyponatremia-sensitive” volume management may be feasible in real-world clinical settings, albeit conditionally.

Specifically, workflow compatibility was viewed as an important condition for promoting consistent implementation in healthcare professionals. This focus aligns with implementation science frameworks that emphasize “fit” with existing workflows as a key determinant of adoption and sustainment [29]. Participants noted that in busy inpatient settings, if individualized targets add extra steps or documentation burden, they are prone to being simplified in practice into generic low-sodium and fluid restriction advice, thereby undermining consistency. A pattern that mirrors broader evidence that documentation burden and workflow misalignment can increase cognitive load and drive workarounds [30]. In contrast, participants perceived implementation to be more workable when hyponatremia-sensitive individualized targets were embedded within existing heart failure care pathways, such as admission assessment, ward rounds, discharge planning, and follow-up linkage. Standardized documentation and patient-facing tools were viewed as helpful supports. Examples included discharge-aligned electronic medical record templates, intake and output logging with reminder functions, and translating bedside education into a home checklist. Collectively, these approaches were perceived as potentially reducing execution difficulty, clarifying responsibilities at key care points, and strengthening the continuity of information and guidance from hospital to home [31].Taken together, our findings suggest that improving compatibility may require further optimization of documentation and transitional processes, although the specific forms and associated workload warrant assessment across different contexts. This also suggests that hyponatremia-sensitive volume management represents a further development of routine volume management workflows, achieved through greater refinement, clearer task delineation, and more explicit role allocation rather than a departure from conventional practice.

Meanwhile, hyponatremia awareness and belief provided an important motivational and cognitive foundation for implementation. Similar studies have also emphasized the importance of patients’ illness perceptions [32]. In interviews, both patients and clinicians commonly expressed concern about hyponatremia-related risks and felt that recommendations from trusted healthcare professionals could increase willingness to adhere. This emphasis on patient understanding is also consistent with heart failure self-care guidance [33]. In some accounts, heightened risk awareness and recognition of the value of the intervention may prompt volume management to move from the period of advice toward more sustained self-management behaviors. However, participants also noted that recommendations may require stage-specific adjustments in the context of hyponatremia, for instance, different sodium intake requirements at different phases. Healthcare professionals cautioned that without clear explanations and consistent team-wide messaging, patients may become confused and their ability to implement recommendations may be compromised. Accordingly, this facilitator also highlights the need for more coherent, understandable risk communication and greater consistency of information to reduce confusion and support maintenance of behaviors [34]. This suggests that hyponatremia-sensitive volume management has a shared motivational and cognitive foundation across both clinicians and patients. At the same time, it also underscores a higher requirement: the principles and content used to strengthen patients’ understanding and motivation for hyponatremia-sensitive volume management must be sufficiently coherent and consistent, so as not to cause confusion or distress for patients.

In addition, tailored targets and support further underscore that, in the context of hyponatremia, actionable personalization is both a facilitator and a marker of higher requirements [35]. Participants emphasized that volume management was easier to understand and sustain when goal setting aligned with patients’ daily capacity, health literacy, comorbidity burden, and available family support. It should be noted that the present study primarily reflects participants’ experiences and suggestions regarding the need for personalization. Translating these needs into more standardized and operational tools, including goal ranges, adjustment triggers, and stratified support packages, remains a potential direction for subsequent intervention development and implementation research and warrants further validation.

Overall, implementing hyponatremia-sensitive volume management was perceived as acceptable and feasible by patients, family caregivers, and healthcare professionals in our study. Stakeholders believed that a tailored, workflow-embedded volume management approach and strong awareness and beliefs would enhance adherence to volume-related recommendations, strengthen self-management, and were perceived as having the potential to reduce rehospitalization risk and symptom deterioration. These perceptions align with prior evidence suggesting that individualized, patient-centered heart failure volume management strategies, supported by patients’ illness understanding and rehabilitation beliefs, can strengthen adherence to volume management and translate into better clinical outcomes [36–39].

### Barriers: strategies to optimize volume management in patients with heart failure and hyponatremia

Hyponatremia-sensitive volume management was often perceived by participants as a higher clinical requirement in heart failure care, potentially pointing toward a more personalized, precise, and dynamic direction for volume management, which is consistent with previous studies [40].

Based on the synthesized barriers, this study highlighted potential strategies to support hyponatremia-sensitive volume management at multiple levels.

#### Patient and caregiver level: education tailored to complexity

At the patient and caregiver level, they show the awareness and understanding of the importance of hyponatremia-sensitive volume management, such as the harms of hyponatremia and the importance of volume management. However, they also reported clear difficulties in handling complex instructions, which mirrors recent qualitative work highlighting substantial self-care challenges for patients and family caregivers despite high motivation to participate in heart failure management [41, 42]. Patients and family carers were motivated to manage volume, yet many had difficulty with millilitre- and gram-based calculations, interpreting weight changes, and judging when to seek help, which aligns with prior studies suggesting that frailty, cognitive burden, and limited health literacy undermine day-to-day heart failure self-care behaviours [43, 44]. This points to a need for simple, hyponatremia-sensitive educational tools designed for older, multimorbid patients and their caregivers, such as pictorial salt-intake guides, colour-coded weight charts and concrete examples of “safer” versus “riskier” fluid and sodium patterns, in line with patient-reported preferences for practical, realistic and situation-specific self-care support, as suggested in prior qualitative work from patient perspectives [42]. Presenting volume management in the language of daily routines rather than abstract numbers is consistent with previous work showing that heart failure self-care improves when education and interventions are tailored to patients’ cognitive, functional and social circumstances and when key behavioural determinants such as perceived control, self-efficacy and grit are addressed [45, 46]. Qualitative and interventional studies on heart failure self-care similarly stress that practical, ongoing, often nurse-led support is particularly important for older patients with multiple conditions and high treatment burden [47]. From an implementation science perspective, this study is to show that patients and caregivers have a clear awareness of hyponatremia-related risks, yet implementation still requires practical, actionable knowledge and supports to translate this foundation into sustained hyponatremia-sensitive volume management.

#### Clinical team level: operationalising individualized plans through roles and skills

At the level of the clinical team, the main issue from the healthcare professionals is how to make individualized plans workable in everyday practice. Our findings indicate that clinicians are generally confident in evidence-based, hyponatremia-sensitive strategies. They recognize a clear division of roles, with physicians acting as planners and nurses as practitioners. This is consistent with evidence that multidisciplinary cardiovascular and heart failure programmes rely on clearly defined nurse and physician roles to deliver complex care [48, 49]. Individualized volume management was described as cognitively demanding and, under time pressure, was often reduced to generic “low-salt, low-fluid” advice, echoing survey data showing substantial variation in nurses’ heart failure knowledge and the need for targeted, practice-oriented education [50].

These findings suggest team-focused strategies may benefit from prioritizing operationalisation, including tools, workflows, skills, alongside supporting readiness and sustained engagement where needed. Related reviews showing that structured nursing interventions and transitional care models are most effective when they provide concrete tools, protocols and follow-up [51]. Short, case-based training on interpreting sodium trends, titrating fluid and sodium targets, and setting realistic goals, together with making the “physician as strategist, nurse as implementer” model explicit in protocols and handover tools, may support more consistent bedside practice [52].

#### Organizational and system level: tools, coordination and community support

At the organizational and system level, participants described limited readiness, lack of simple visual tools, fragmented communication across care settings and barriers such as drug costs and travel distance, consistent with reviews showing that system resources, coordination and access shape heart failure self-care [53–55]. These conditions blunt the impact of patient education and clinical team efforts, so system-oriented strategies need to prioritise shared tools and links between services. Concise order sets for fluid and sodium targets, standard templates for individualized prescriptions and brief follow-up checklists may reduce reliance on personal experience and shift-to-shift variation, in line with studies indicating that structured discharge protocols and standardised forms improve teaching consistency [56, 57]. Embedding these tools in shared records across wards, clinics and community services could strengthen continuity of care for patients with hyponatremia and reflects evidence that nursing and transitional-care programmes work best within coordinated pathways [58, 59]. Where high drug costs, travel and limited community resources impede implementation, collaboration with primary care and community nurses, plus low-threshold follow-up such as structured calls or basic telemonitoring, may help maintain volume management nearer to patients’ homes [60].

### Strengths and limitations

This study provides a CFIR 2.0–informed, multi-stakeholder account of facilitators and barriers to implementing hyponatremia-sensitive volume management in heart failure, incorporating perspectives from patients, family caregivers, nurses, and physicians across hospitalization and the early post-discharge transition. These findings offer practice-facing insights that may inform role-specific implementation strategies for individualized, hyponatremia-sensitive volume management.

Several limitations should be considered when interpreting the findings and their transferability. First, as the study was conducted in a single tertiary hospital, workflow-related facilitators and barriers may reflect local staffing, documentation systems, and routines; thus, transferability may be greatest to similar inpatient settings, and caution is needed when extrapolating to primary care, community follow-up, or less-resourced hospitals. Second, the relatively short data-collection window may have emphasized near-term hospitalization and discharge experiences, potentially under-capturing how barriers and self-management challenges evolve over time. Third, the absence of system-level stakeholders, such as policymakers and community providers, may have limited “outer setting” insights relevant to scale-up, including reimbursement, community resource availability, and cross-institutional coordination.

The number of participants per stakeholder group was modest compared with some qualitative studies that include 16–20 interviews per group. However, fewer interviews may be sufficient when the study has a narrow and specific research aim, a relatively homogenous clinical context, and data are generated through in-depth interviews with a focused semi-structured guide. In our study, interviews were designed to elicit detailed accounts and were complemented by concurrent analysis and iterative probing, which likely increased interview yield and accelerated saturation. Nevertheless, we acknowledge that additional interviews, particularly across more diverse sites and healthcare systems, may have identified less common perspectives; future multi-site studies with broader sampling are warranted to test the transferability of these findings.

These limitations inform future research. Multi-site and longitudinal studies that include community and primary-care stakeholders are needed to test feasibility and acceptability across contexts and to identify which workflow tools, communication approaches, and follow-up models best sustain hyponatremia-sensitive volume management in heart failure over time.

## Conclusions

Hyponatremia-sensitive volume management emerged as a more personalized, precise, and dynamic extension of routine heart failure volume care rather than a competing paradigm. The main implementation challenge lay not in motivation, but in a persistent mismatch between complex regimens and older patients’ everyday capabilities and support conditions, indicating that scalable impact requires simplified tailored education, workflow-embedded plans, and strengthened transitional and community supports.

## Supplementary Information

Supplementary Material 1.

Supplementary Material 2.

## Data Availability

The data that support the findings of this study are available from the corresponding author upon reasonable request.

## Abbreviation

CFIR 2.0: Consolidated Framework for Implementation Research, version 2.0
