# Supplementary material for: Facilitators and barriers to volume management interventions in patients with heart failure and hyponatremia: a qualitative study
Source: BMC Geriatr. 2026 Feb 2;26:287. doi: 10.1186/s12877-026-07080-y (PMC12952140; doi:10.1186/s12877-026-07080-y)
Supplement: Supplementary file 1 — Supplementary Material 1. [file 12877_2026_7080_MOESM1_ESM.docx]

**医生访谈提纲**

1. 您认为心力衰竭患者发生低钠血症有什么危害性？对该类患者进行容量管理有哪些措施？（个体的动机）
2. 哪些具体措施在临床执行中最难落实？您认为主要原因是什么？比如江西所处地域的饮食文化、经济水平、社区医院、生活习惯、在人力、设备、资金、流程、领导支持、政策等因素？
3. 针对这些不足，我们可以从哪些方面去完善呢？
4. 您认为您在患者的容量管理中我们医生承担何种任务？ 我们还需补充哪些相关知识或技能？（个体的能力、机会）
5. 目前您在临床采取的对心衰伴低钠血症患者进行容量管理的方案，您们在临床诊断与治疗中，有采用哪些相关的指南或专家共识支持？（创新的证据基础）
6. 方案开展后，您认为我们可以通过哪些指标或方式评估效果并持续质量改进？（反思与评价）

**护士访谈提纲**

1. 在您的日常工作者，是否有关注到心力衰竭伴低钠血症这部分患者？您认为自己在患者的容量管理中担任何种角色？（动机）
2. 与心力衰竭人群普遍进行“低盐低钠”的健康管理理念相比，您认为如何对并发“低钠血症”的患者实施个体化、针对性的容量管理措施？（能力、机会）
3. 您知道有哪些正在实施的对心衰伴低钠血症患者的容量管理措施（比如出入量记录）？您是否接受过基于最新临床证据（如指南更新、研究成果）的专项培训？(创新的来源、适应性及适用性）科室在心衰伴低钠血症患者容量管理中配置了哪些护理资源（如专科护士数量、监测设备、教育材料、电子系统等）？还需补充哪些资源？（可用资源、知识和信息的可及性、物质基础）
4. 从患者入院评估到出院随访，容量管理的护理流程是什么？科室质量控制小组在容量管理方面起什么作用？质控指标的设定与执行情况如何？您认为可以采用哪些评价指标（工作基础、协作沟通、创新对现有流程的兼容性）
5. 实施过程中有什么常见的误区？可以如何改善（反思与评价）

**照护者访谈提纲**

1. 您清楚患者患病后在生活上有哪些注意事项吗（比如限水限钠、管理体重）？知识来源是什么？（创新来源、个体能力）
2. 您认为这些方法对于患者病情有哪些影响?（个体动机）
3. 您在家如何照顾患者，承担了哪些照护工作？您能说出一天的照护流程吗？（实施过程执行）
4. 家里会准备哪些工具或设备帮助患者管理容量呢？您觉得好用的有哪些？是否有改进建议？（内部因素-材料和设备）
5. 长期照护患者过程中，遇到哪些困难（例如患者不配合、不清楚正确的操作流程）？您是如何解决的？（实施过程-反思与评价）
6. 您知道判断患者病情缓解或加重的方法吗？通过什么判断？（个体能力、实施过程-反思与评价）
7. 患者出现容量管理相关问题（如体重骤增、水肿）时，您会如何处理？寻求帮助的渠道有哪些（例如医院、社区护士）？（外部因素-资源）

**患者访谈提纲**

1. 基本资料：您首次被诊断为心力衰竭的时间、既往因心衰或低钠血症住院/急诊次数
2. 您听说过“液体管理”这个说法吗？您知道“液体管理”具体要做哪些事吗？这些知识来源是什么？（创新来源、个体能力）
3. 您觉得自己 “液体管理” 的能力怎么样？比如您清楚每日应该喝多少水、吃多少盐、称体重的正确方法吗？（个体能力）
4. 具体谈谈您是怎么做的？比如喝水、称体重的方法？用到了哪些工具协助“液体管理”（控盐勺、刻度杯）？（实施过程-执行、内部因素-材料和设备）
5. 您知道自己用的利尿剂名字吗？用利尿剂过程中您如何判断效果？会自行调整剂量吗？（个体能力、实施过程-调整策略）
6. 在患病之前您的饮食口味如何？平时喜欢运动吗？患病之后需要限盐限水，和之前的习惯是否冲突？如果冲突，您是如何协调的？（个体动机、实施过程-适应）
7. 您觉得注意限盐限水后，您的身体有什么具体变化吗？（个体特征-需要、实施过程-反思与评价）
8. 您平时和谁住一起？家属是否协助您管理体重？具体如何帮助您的？（外部因素）
9. 您居家遇到液体管理方面的问题时，您会通过哪些渠道解决问题？（外部因素-资源）
10. 出院后居家管理时，有接到医院的随访电话吗？具体会问您些什么问题（比如体重管理的情况、提醒您每周去复查）？（内部因素-资源、实施过程-反思与评价）
11. 住院或复诊时，医生护士会专门教您容量管理吗？比如教您如何记录出入量、看体重变化？（实施过程-反思与评价）
12. 在医院治疗时，您觉得医护人员对“液体管理”重视吗？查房时会询问您饮水量和体重吗？（实施过程-反思与评价）
13. 您住的社区有没有组织教心衰患者测体重、正确喝水、控制盐摄入、进行运动锻炼类似这样的活动？如果没有，对于这方面您有什么体会或建议？（外部因素）
