# Supplementary material for: Facilitators and barriers to volume management interventions in patients with heart failure and hyponatremia: a qualitative study
Source: BMC Geriatr. 2026 Feb 2;26:287. doi: 10.1186/s12877-026-07080-y (PMC12952140; doi:10.1186/s12877-026-07080-y)
Supplement: Supplementary file 2 — Supplementary Material 2. [file 12877_2026_7080_MOESM2_ESM.docx]

**Interview Guides (English Translation)**

**Physician Interview Guide**

**Version 1**

- In your opinion, what harms or risks does hyponatremia pose to patients with heart failure? What measures are used to manage volume status in this population? (Motivation)
- Which specific measures are the most difficult to implement in routine practice? What do you think are the main reasons (e.g., local dietary culture in Jiangxi, socioeconomic level, community hospital capacity, lifestyle habits; and factors such as staffing, equipment, funding, workflow, leadership support, policies, etc.)?
- Given these gaps, in what ways do you think the current practice could be improved?
- What responsibilities do you think physicians should take on in patients’ volume management? What additional knowledge or skills do you think physicians need? (Capability and opportunity)
- In your current practice for managing volume status in HF patients with hyponatremia, which guidelines or expert consensuses do you rely on to support diagnosis and treatment? (Evidence base of the innovation)
- After implementing a volume management plan, what indicators or approaches would you suggest to evaluate effectiveness and support continuous quality improvement? (Reflection and evaluation)

**Revised Version**

- (1) In clinical practice, patients with HF and hyponatremia may present with a range of adverse symptoms (e.g., fatigue, poor appetite, confusion, refractory edema). Do you pay particular attention to this patient group? When monitoring serum sodium for these patients, what volume management measures do you usually take?
- (2) Regarding current volume management for HF patients with hyponatremia, which steps or links in the process are most difficult to carry out? What do you think are the main reasons?
- (3) How do you think these difficulties could be addressed or improved?
- (4) What role should physicians play in volume management? In what ways can physician collaboration and support better facilitate volume management?
- (5) Concerning existing guidelines and expert consensuses for HF patients with hyponatremia, how would you rate the level/strength of evidence and recommendations? How feasible are they in real-world practice?
- (6) We have not yet established a standardized volume management protocol specifically for HF patients with hyponatremia. Considering dietary culture, socioeconomic status, hospital context, lifestyle habits, hospital management, and clinical practice, what would a more reasonable, personalized, precise, and implementable protocol look like?
- (7) After implementation, what indicators or evaluation methods would you recommend to assess implementation effectiveness?

**Nurse Interview Guide**

- In your daily work, have you noticed or paid attention to patients with heart failure complicated by hyponatremia? What role do you think you play in patients’ volume management? (Motivation)
- Compared with the general “low-salt/low-sodium” health management approach used for many HF patients, how do you think individualized and targeted volume management should be delivered for patients with concomitant hyponatremia? (Capability and opportunity)
- What volume management measures for HF patients with hyponatremia are currently being implemented (e.g., intake–output recording)? Have you received any dedicated training based on the latest clinical evidence (e.g., guideline updates, research findings)? (Source/adaptability and applicability of the innovation)
- What nursing resources are available in your unit for volume management in HF patients with hyponatremia (e.g., number of specialist nurses, monitoring devices, education materials, electronic systems)? What additional resources are needed? (Available resources; access to knowledge and information; physical infrastructure)
- From admission assessment to discharge follow-up, what is the nursing workflow for volume management? What role does the unit’s quality control team play? How are quality indicators defined and implemented? What indicators do you think could be used to evaluate practice? (Implementation context; collaboration and communication; compatibility with existing workflow)
- What common misconceptions occur during implementation, and how could they be improved? (Reflection and evaluation)

**Caregiver Interview Guide**

- Do you understand what patients should pay attention to in daily life after diagnosis (e.g., fluid restriction, sodium restriction, weight monitoring)? Where did you learn this information? (Source of the innovation; individual capability)
- In your view, how do these measures affect the patient’s condition? (Motivation)
- How do you care for the patient at home? What caregiving tasks do you take on? Can you describe a typical day’s caregiving routine? (Implementation process—execution)
- What tools or equipment does your family prepare to help the patient manage volume status? Which are useful, and what improvements would you suggest? (Inner setting—materials and equipment)
- During long-term caregiving, what difficulties have you encountered (e.g., patient non-adherence, uncertainty about correct procedures), and how did you address them? (Implementation process—reflection and evaluation)
- Do you know how to judge whether the patient’s condition is improving or worsening? What cues or methods do you use? (Individual capability; reflection and evaluation)
- When the patient experiences volume-management-related problems (e.g., sudden weight gain, edema), what do you do? What channels do you use to seek help (e.g., hospital, community nurses)? (Outer setting—resources)

**Patient Interview Guide**

- Basic information: When were you first diagnosed with heart failure? How many times have you been hospitalized or visited the emergency department due to heart failure or hyponatremia?
- Have you heard the term “fluid management”? Do you know what “fluid management” involves? Where did you learn this information? (Source of the innovation; individual capability)
- How would you rate your ability to manage fluids? For example, do you know how much water you should drink each day, how much salt you should consume, and the correct way to measure weight? (Individual capability)
- Can you describe specifically how you do it—for example, how you drink water and how you weigh yourself? What tools do you use to support “fluid management” (e.g., salt-control spoon, measuring cup)? (Implementation process—execution; inner setting—materials and equipment)
- Do you know the name of your diuretic? When using diuretics, how do you judge whether they are working? Do you adjust the dose on your own? (Individual capability; implementation process—adaptation)
- Before you became ill, what was your usual taste preference (e.g., salty foods)? Did you exercise regularly? After the illness, you may need to restrict salt and fluids—does this conflict with your previous habits? If so, how do you reconcile these conflicts? (Motivation; implementation process—adaptation)
- After paying attention to salt/fluid restriction, what specific changes have you noticed in your body? (Needs; reflection and evaluation)
- Who do you live with? Do family members help you manage your weight? How do they help? (Outer setting)
- When you encounter problems with fluid management at home, what channels do you use to solve them? (Outer setting—resources)
- After discharge, did you receive follow-up calls from the hospital? What did they ask you about (e.g., weight management, reminding you to return for review)? (Inner setting—resources; reflection and evaluation)
- During hospitalization or follow-up visits, do physicians or nurses provide specific teaching about volume/fluid management, such as recording intake–output or monitoring weight changes? (Implementation process—reflection and evaluation)
- During hospital treatment, do you feel healthcare professionals take “fluid management” seriously? During rounds, do they ask about your water intake and weight? (Implementation process—reflection and evaluation)
- In your community, are there any activities that teach HF patients how to weigh themselves, drink water appropriately, control salt intake, and exercise? If not, what are your thoughts or suggestions? (Outer setting)
